# Efficacy of Chitosan Oligosaccharide Combined with Cold Atmospheric Plasma for Controlling Quality Deterioration and Spoilage Bacterial Growth of Chilled Pacific White Shrimp (*Litopenaeus vannamei*)

**DOI:** 10.3390/foods12091763

**Published:** 2023-04-24

**Authors:** Mijia Yu, Yixuan Ding, Qi Du, Yueqin Liao, Wenhua Miao, Shanggui Deng, Patrick J. Cullen, Rusen Zhou

**Affiliations:** 1 Department of Food Science and Pharmaceutics, Zhejiang Ocean University, Zhoushan 316022, China; 2 School of Chemical and Biomolecular Engineering, University of Sydney, Sydney, NSW 2006, Australia

**Keywords:** combined treatment, quality properties, microbiota composition, chilled storage, *Litopenaeus vannamei*

## Abstract

A novel food processing technique based on the combination of cold atmospheric plasma (CAP) and chitosan oligosaccharide treatment (COS) was developed to enhance antibacterial performance and extend the shelf life of Pacific white shrimp (*Litopenaeus vannamei*). Effects of different treatments on the microbial community composition, physicochemical properties, and post-storage behaviors of Pacific white shrimp were evaluated during chilled storage for up to 10 days. Results showed that the synergistic effects of COS and CAP could be obtained, largely inhibiting the growth of microorganisms. The content of total volatile basic nitrogen (TVB-N), total viable counts (TVC), and pH value in treated groups were lower than in the control group and the loss of moisture content, water activity, and sensory score were observed. Compared to the control group, shrimp was on the verge of spoilage on the 6th day of storage, while the COS–CAP-treated shrimp had a 4-day lag period. Moreover, the COS and CAP could effectively inhibit the growth of *Aliivibrio*, the predominant microbial group in the ultimate storage period. This study suggests that the combined utilization of COS and CAP could be a high-efficacy technique for extending the shelf-life of shrimp.

## 1. Introduction

Due to its economic worth, appealing flavor, and high nutritional content, Pacific white shrimp (*Litopenaeus vannamei*) has been a globally popular and widely disseminated fishery product with growing market demands [1]. Recognized as having outstanding protein, Pacific white shrimp are also known to have well-balanced amino acids, and premium functional lipids [2]. Consumers today are becoming increasingly attentive to the security and freshness of aquatic items. However, shrimps are very perishable and textural degradation occurs as a consequence of the combined impacts of enzymatic proteolysis and microbial metabolism during postharvest preservation and the retail period, leading to geographical and seasonal constraints on production and sale [3]. Traditional preservation techniques, including freezing, superchilling, and refrigeration, are still widely employed in today’s market. Utilizing the new preservation technologies for commercial purposes raises several issues and restrictions, such as the potential risks of interactions between packaging materials and shrimp products and the detrimental effects on flavor and taste caused by lipids, plant extracts, and essential oils [4]. Thus, combining two or three practical and effective preservation techniques has attracted lots of attention as a “hurdle preservation technique”, to maintain the shelf life of shrimp.

Cold plasma, the fourth physical state developed at ordinary temperature, which consists of several reactive species including photons, electrons, positive and negative ions, free radicals, molecules, and atoms of the different excitation states [5,6]. These species show good bacteria inhibition characteristics, making CAP (cold atmospheric plasma) a suitable preservation strategy. Additionally, CAP has been effectively researched for food decontamination [7], including the inactivation of pathogens, spores, and bacteria [8]. Antimicrobial actions are the consequence of a synergistic contribution from all plasma species that may harm the outer surface of microbial cells and biological stuff such as lipids, proteins, DNA, and cytoplasm without affecting dietary qualities. Moreover, in-package plasma processing has shown encouraging results in preserving the nutritional and quality attributes of food goods during treatment and throughout their shelf life [9] and CAP has been used effectively to extend the shelf life of food. Notably, the antibacterial effectiveness of CAP is dependent upon a number of variables including frequency, voltage, working gas, and treatment time [10]. However, the presence of reactive species can be more prone to oxidative deterioration, accelerating the deterioration of food and influencing flavor. Thus, the suitable combination of preservatives or antioxidants should be selected for optimal CAP formation and other hurdle technologies to optimize the preservation efficacy of the treated food throughout storage.

Chitosan oligosaccharide (COS), a naturally occurring oligosaccharide derived from chitosan degradation [11], represents a class of polymers that have grown in popularity in the culinary, agriculture, and cosmetics industries, which, in addition to its beneficial qualities including its multiple nontoxic, antibacterial, antioxidant, reduced viscosity, biocompatible, and biodegradable capabilities, is also water-soluble and low in viscosity. [12,13]. COS exhibits excellent antioxidant activity in vitro and in vivo [14]. The available hypotheses generally concentrate on two points of view when it comes to the antibacterial mechanism of COS: the positively charged COSs interact with negatively charged bacterial surface groups, altering membrane stability and increasing microbial death [15]; as a low-molecular-weight glycan, COS directly penetrates the membrane and reacts with the interior cytoplasm, disrupting the normal metabolic processes of bacteria [16]. Moreover, several studies have demonstrated that COS has the capacity to inhibit many bacteria and fungi, making it a promising natural antibacterial agent [17]. In addition, COS performs an antioxidant activity, preventing the oxidative damage of shrimp and other aquatic foods caused by CAP [14,18].

This study aimed to extend the shelf-life of chilled Pacific white shrimp utilizing CAP and COS as two nonthermal processing techniques. Effects on the shelf life of shrimp treated by CAP and COS were identified based upon the changes in sensory scores and spoilage indicators, including moisture content, water activity, morphology, color, odor, texture, weighted sensory score, total volatile basic nitrogen (TVB-N), pH, and histological analysis. In addition, Illumina-MiSeq high-throughput sequencing was used to examine the changes in the microbiota composition of samples during chilled storage.

## 2. Materials and Methods

### 2.1. Sample Preparation

Six hundred fresh Pacific white shrimp (55–60 pieces/kg, Zhoushan Xincheng market) were supplied to the laboratory alive with oxygen inflating packing bag. Later, Pacific white shrimp were killed by submerging them for 20 min in an ice-water slurry with a 3:1 (*w*/*v*) ice/water ratio and randomly assigned into four groups: C (untreated samples), COS (samples treated with 1% (*w*/*v*) chitooligosaccharide), CAP (samples treated with cold atmospheric plasma (50 kV, 4 min)), and COS–CAP (samples treated with 1% (*w*/*v*) chitooligosaccharide firstly and atmospheric cold plasma (50 kV, 4 min) later). For COS group and COS–CAP group, shrimp were immersed in an COS solution for 30 min at 4 °C before packed individually into CT-PP containers measuring 210 × 135 × 35 mm. Then, the trays were sealed using a MAP machine (MAP-H360, Senrui Fresh-keeping Equipment Co., Ltd., Suzhou, Jiangsu, China). For CAP group and COS–CAP group, shrimp were treated with a dielectric barrier discharge (DBD) plasma (Phenix BK130/3 AC Test Set 600 Series Processor, Phenix Technologies, Inc., Accident, MD, USA) after being packeted [19], and CT-PP containers containing shrimp samples were positioned between two parallel rounded aluminum plates with a 155 mm outside surface and a 75 mm spacing between the two electrodes [20]. During storage, at 4 ± 0.5 °C, shrimp samples were randomly chosen for measurement every two days.

### 2.2. Sensory and Color Analysis

According to Zhao et al. [21], a well-trained team of ten individuals with sensory evaluation experts conducted the sensory analysis with slight modification. The morphology, odor, color, texture, and weighted sensor were assessed on a 10-point scale (1–2 = severe spoiled, 3–4 = clear defect, 5–6 = mild/slight defect, 7–8 = satisfactory, 9–10 = fresh/admirable). When the median grade received was 4 or below, the sample was deemed spoilt. For each group, three cartons of shrimp were randomly chosen to assess their organoleptic qualities.

The color properties (including brightness: L*; red–green index: a*; and yellow–blue index: b*) were determined in shelled shrimps (surface muscle, central part) from each group using an automatic CR-400 colorimeter (Konica Minolta Investment Ltd., Shanghai, China) carried out according to Jiao et al. [22].

### 2.3. Moisture Content and Water Activity (A_W_) Analysis

The moisture content was measured according to the National Food Safety Standard Method of China (Determination of moisture content in food; GB, 5009.3-2016). The A_W_ value was measured using a Fast-lab water activity meter (JC-HD, Qingdao Juchuang Environmental Co., Ltd., Qingdao, China) at room temperature.

### 2.4. Determination of TVC, TVB-N, and pH Value Analysis

TVC (total viable counts) were measured according to the method described by Jia et al. [23]. For each sterile bag, 5 g cracked shrimp muscle was homogenized with 45 mL sterile 0.9% NaCl (*w*/*v*) solution. A saline solution diluent was used to create further serial dilutions from the homogenate. The total viable count was determined using appropriate dilutions to spread on a plate count agar and then enumerated after incubating at 30 °C for 72 h.

The TVB-N value of the chilled-stored sample was determined using a steamed distillation method according to Jiao et al. [22]. Ten-gram minced samples were mixed with 70 mL of deionized water, then soaked for 30 min and mixed with 1 g of light magnesia. After that, analysis was conducted using an Automatic Kjeldahl Apparatus (KDN-520, Bangyi Precision Meter (Shanghai) Co., Ltd., Shanghai, China). Results were expressed as mg N per 100 g flesh.

A digital pH meter was used to measure the pH value (PHS-3c, Shanghai Yidian Scientific Instrument Co., Ltd., Shanghai, China). Briefly, 3 g of shrimp flesh was dispersed in 27 mL normal saline and homogenized for 2 min. The homogenate was centrifuged under the appropriate condition (3000× *g*, 10 min); then, the supernatant was gathered and measured.

### 2.5. Histological Analysis

The hematoxylin and eosin (H&E) staining method described by Zhang et al. [24] was employed to identify the histological alterations that occurred in the muscle tissue of the shrimp. Firstly, the shrimp samples were cut into slices (5-milimeters-thick) and fixed in LEAGENE’s 4% PFA; then, a graded succession of ethanol solutions was used to dehydrate. After dehydration, the sample tissues were fixed in paraffin and cut into 5 m transverse sections using a microtome. The tissue sections were then H&E-stained and histologically examined (model CX23, Olympus Co., Ltd., Beijing, China) using a light microscope (Olympus Co., Ltd., Beijing, China). Microstructural changes were recorded.

### 2.6. Microbiota Composition Analysis Based on Illumina-MiSeq Sequencing

According to the manufacturer’s recommendations, total microbial genomic DNA was isolated from shrimp samples using the FastDNA^®^ Spin Kit (MP Biomedicals, Santa Ana, CA, USA). Prior to further usage, the DNA quality and concentration were assessed using a NanoDrop^®^ ND-2000 spectrophotometer and 1.0% agarose gel electrophoresis from Thermo Scientific Inc., Waltham, MA, USA. Using primer pairs 338F (5′-ACTCCTACGGGAGGCAGCAG-3′) and 806R (5′-GGACTACHVGGGTWTCTAAT-3′) [25], the hypervariable region V3–V4 of the bacterial 16S rRNA gene was amplified (ABI, Los Angeles, CA, USA). Quantities of 0.8 mL of each primer (5 M), 0.4 mL of Fast Pfu polymerase, 10 ng of template DNA, and ddH2O were added to a total volume of 20 mL of the PCR reaction mixture. Three copies of each sample were amplified. The PCR product was extracted from 2% agarose gel by the AxyPrep DNA Gel Extraction Kit (Axygen Biosciences, Union City, CA, USA), then purified as directed by the manufacturer, and quantified using a Quantus^TM^ Fluorometer (Promega, San Luis Obispo, CA, USA).

Majorbio Bio-Pharm Technology Co. Ltd., (Hangzhou, China) pooled purified amplicons in equimolar proportions and performed paired-end sequencing on an Illumina MiSeq PE300 platform (Illumina, San Diego, CA, USA) following the recommended protocols (Shanghai, China). The NCBI Sequence Read Archive (SRA) database received the raw sequencing reads. Raw FASTQ files were demultiplexed using an internal Perl script, and then FASTP version 0.19.6 [26] and FLASH version 1.2.7 [27] were used to merge and quality-check them. UPARSE 7.1 [28,29] was used to cluster the optimized sequences into operational taxonomic units (OTUs) with a 97% sequence similarity threshold. Each OTU’s most prevalent sequence was chosen as the representative sequence. Each sample’s 16S rRNA gene sequence count was decreased to 20,000, which yet received a 99.09% average Good’s coverage, to reduce the effects of sequencing depth on alpha and beta diversity measures. RDP Classifier version 2.2 [30] used a confidence threshold of 0.7 to compare each OTU representative sequence’s taxonomy to the 16S rRNA gene database (for example, Silva v138). The entire analysis process followed PICRUSt2 protocols.

### 2.7. Data Analysis

All measurements except for color properties were made in triplicate and expressed as mean ± SD. The least significant difference (LSD) procedure was used to compare different groups using SPSS 25.0 software (SPSS Inc., Chicago, IL, USA). Analysis of variance was performed between means to determine the significant differences using Duncan’s multiple range test. Pearson correlation analysis was performed between microbiota composition and physiochemical parameters. A value of *p* < 0.05 was considered statistically significant.

## 3. Results and Discussions

### 3.1. Microbial Analysis

#### 3.1.1. Total Viable Counts (TVC) Analysis

**Figure 1:**
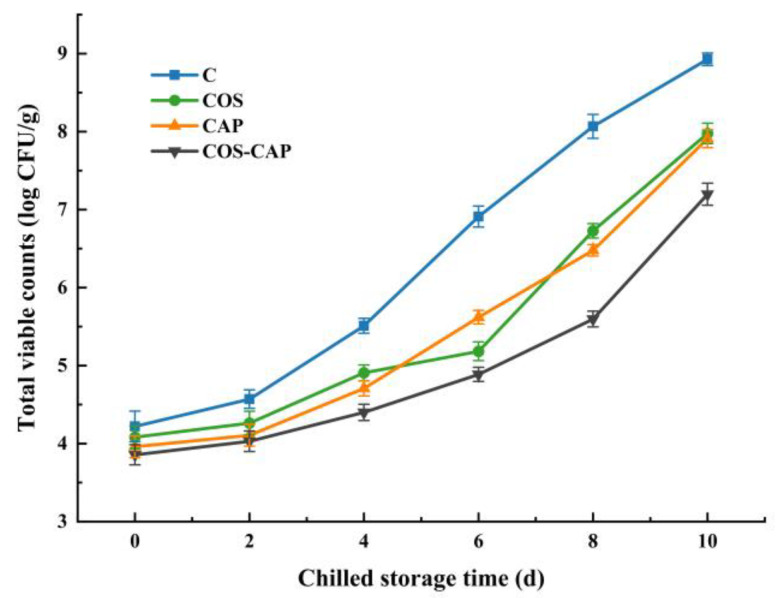
Changes in TVC of Pacific white shrimp during storage at 4 °C in control (C), 1% COS-treated group (COS), cold atmospheric plasma (50 kV, 4 min)-treated group (CAP), and 1% COS combined with CAP-treated group (COS–CAP).

The TVC is an important element in determining the freshness of shrimp samples. The initial TVC of C, COS, CAP, and COS–CAP groups was 4.22, 4.08, 3.96, and 3.86 log CFU/g, respectively (Figure 1). The microbial growth was positively correlated to the storage time, with the most rapid increase observed in the C group. The TVC of samples untreated and treated with COS, CAP, and COS–CAP overpassed the acceptance limit (7-log CFU/g) on days 7, 9, and 10, respectively, which demonstrated that COS and CAP were both efficient at delaying microbial growth. COS combined with CAP treatment showed the more obvious effect of extending the shelf-life of chilled Pacific white shrimp. The ionized gas produced by CAP consists of reactive species, including reactive nitrogen species (RNS) and reactive oxygen species (ROS); free radicals, charged particles, molecules, and atoms in their ground and excited states, along with photons [5]; which might actuate the scavenging activity to facilitate inactivation of microbial cells. Regarding the COS, positively charged COSs interact with negatively charged groups on the surface of bacteria, disrupting membrane stability and inducing microbial death. Conversely, as low-molecular-weight glycans, COSs directly permeate the membrane and react with the interior cytoplasm, disrupting the normal metabolic processes of microbes [17]. Therefore, the combination of COS and CAP effectively led to the death of microorganisms.

#### 3.1.2. Richness and Diversity of the Microbial Community

The microbiota composition of shrimp samples during storage was investigated using high-throughput sequencing on the Illumina MiSeq platform. A total of 1,385,833 high-quality sequencing reads were obtained after quality filtering. The results of alpha diversity (Table 1) show that the coverage of all groups was 99%, meaning that the majority of the microbial phylotypes were detected. During the 10 days’ chilled-storage, the OTUs of C group decreased from 151 to 53, COS group from 157 to 54, CAP group from 169 to 29, and CC group from 115 to 35. Meanwhile, it was lower than the Chao1 and ACE index, well consistent with the study by Lan et al. [31]. The high value of ACE and Chao demonstrated the high bacterial community richness [32]. The values of the Chao1 and ACE declined in all treatments with increasing storage time, which indicated that the richness of the microbial community in sample of shrimp declined significantly. Furthermore, the Shannon and Simpson indices representing the species diversity decreased after 8 days of storage. In addition, the values of Shannon in treated samples (including COS, CAP, COS–CAP) were higher than control samples on the 4th and 8th days of storage. The result indicates that COS group and CAP group are more effective than the control group at reducing microbial species in shrimp samples.

#### 3.1.3. Microbial Community Structures

**Figure 2:**
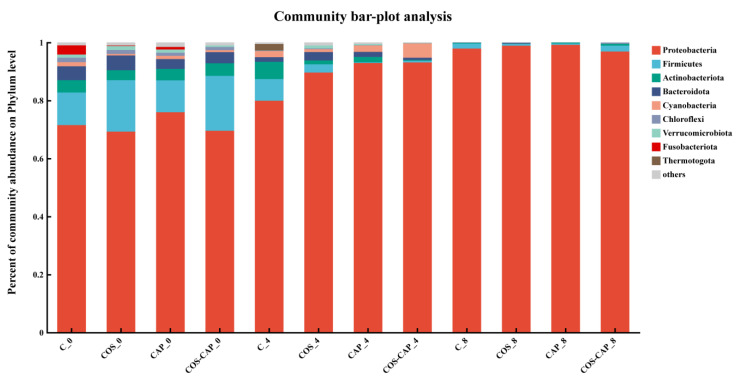

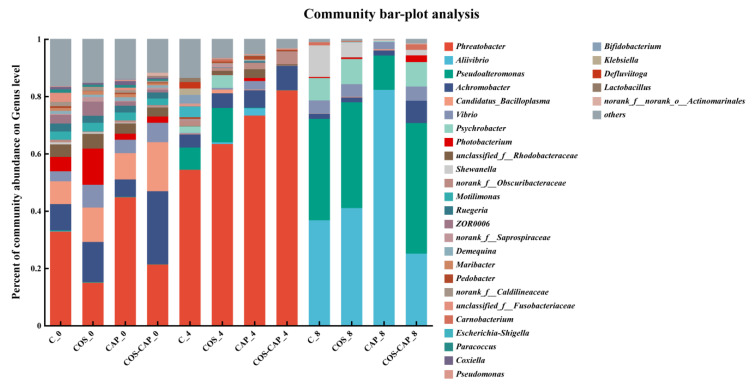
Community bar-plot analysis of microbiota at the phylum level and genus level from Pacific white shrimp during chilled and freeze-chilled storage, in control (C), 1% COS-treated group (COS), cold atmospheric plasma (50 kV, 4 min)-treated group (CAP), and 1% COS combined with CAP-treated group (COS–CAP).

OTUs of the same sort were clustered at the levels of phylum and genus; composition and relative abundance of microbiota at phylum level among all groups are shown in Figure 2. At the beginning of the storage, the dominant phyla were Proteobacteria, Firmicutes, Actinobacteriota, Bacteroidota, and Cyanobacteria. Meanwhile, Proteobacteria was the predominant bacterial genus, which accounted for 69.2–75.9%, and Firmicutes accounted for 11.23% (C), 17.75% (COS), 11.01% (CAP), and 18.91% (COS–CAP) in the initial storage period. Fusobacteriota and Thermotogota were found in low abundances. Firmicutes and Proteobacteria were shown to be the predominant strains in meat and meat products during preservation, according to some studies [33,34]. The relative abundances of Proteobacteria and Cyanobacteria were consistently increased over the growth time, whereas those of Firmicutes, Bacteroidota, Chloroflexi, and Verrucomicrobiota exhibited an opposite pattern. In contrast, the relative abundances of Cyanobacteria initially increased and then decreased afterward. The changes above indicated significant alterations in bacterial community composition over the storage time of shrimp samples.

**Figure 3:**
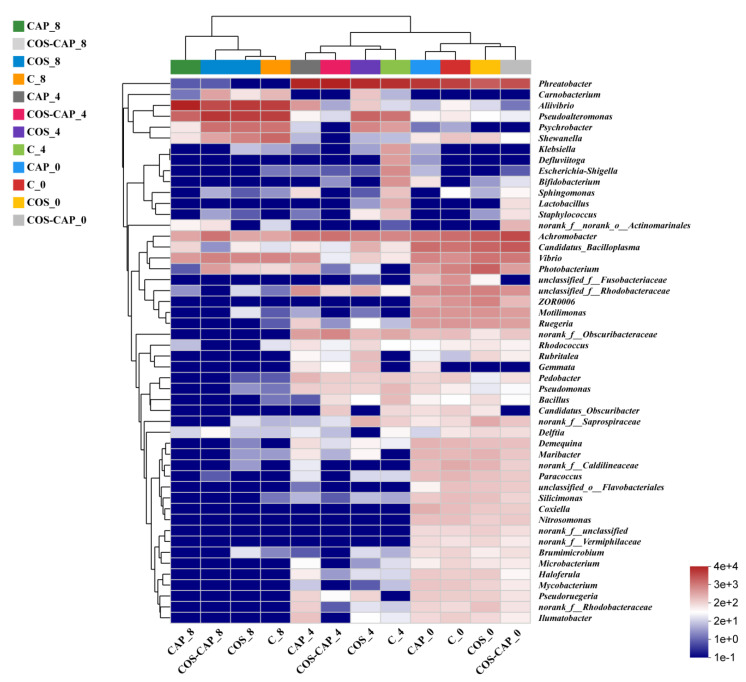
Heatmap analysis of microbiota at the genus level from Pacific white shrimp during chilled and freeze-chilled storage, in control (C), 1% COS-treated group (COS), cold atmospheric plasma (50 kV, 4 min)-treated group (CAP) and 1% COS combined with CAP-treated group (COS–CAP).

The genus-level analysis showed that the composition of bacterial communities changed dramatically in control and treated groups during the storage (Figure 2). It was found that *Phreatobacter*, *Aliivibrio*, and *Pseudoalteromonas* ranked at the top in relative abundance. *Phreatobacter* accounted for the highest proportion in the control group on day 0, followed by *Achromobacter*. With the storage time increasing, the microbial composition changed remarkably. As shown in Figure 3, the bacterial populations dramatically decreased microbial complexity with only a few dominant species at the genus level. The relative abundance of *Phreatobacte*, *Obscuribacteraceae*, and *Rhodobacteraceae* was reduced to 0. At the same time, *Pseudoalteromonas*, *Vibrio*, and *Psychrobacter* were dominant in the bacterial population at the end of storage and became the predominant bacteria. For C, COS, CAP, and COS–CAP groups, the proportion of *Aliivibrio* increased to 36.7%, 41.0%, 82.3%, and 25.1%, respectively, while the proportion of *Pseudoalteromonas* increased to 35.4%, 36.9%, 12.0%, and 45.6%, respectively. The proportion of *Vibrio*, which accounted for a small proportion In C, COS, CAP, and CC, ranged from 2.5% to 5.0%.

As shown in the heatmap (Figure 3), the composition and dynamics of the bacterial community in all samples were analyzed at genus level, and the dominant spoilage bacteria could be observed more easily. The stronger red and blue colors illustrate higher and the lower relative abundance, respectively. In the present study, the microbiota’s community diversity gradually decreased along with time in all four groups, probably due to the growth inhibition in interbacterial competition [35]. The main dominant genera in four group samples of Pacific white shrimp on day 0 and day 4 was *Phreatobactert*, which changed into *Aliivibrio* and *Pseudoalteromonas* on day 8.

### 3.2. Physicochemical Analysis

#### 3.2.1. Water Content and Water Activity (A_W_) analysis

**Figure 4:**
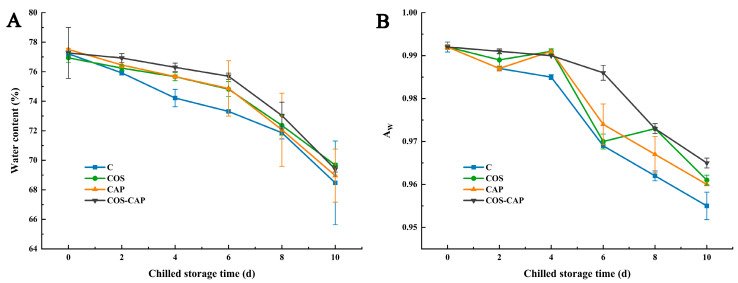
Changes in water content (**A**) and water activity (**B**) of Pacific white shrimp during storage at 4 °C in control (C), 1% COS-treated group (COS), cold atmospheric plasma (50 kV, 4 min)-treated group (CAP), and 1% COS combined with CAP-treated group (COS–CAP).

The water content is closely related to the storage stability of aquatic products, which directly or indirectly affects food quality. The efficacy of COS and CAP treatment on the water content of shrimp was illustrated in Figure 4. The water contents of the four groups decreased gradually and the C group decreased considerably from the 4th day while the COS–CAP group decayed considerably from the 8th day, and on the 10th day, there was no significant difference observed between untreated group and treated group, the values of which were 68.47%, 69.67%, 68.96%, 69.40%, respectively. It is possible that on day 10, all four groups of shrimp were completely corrupted, and the tissue fluid was completely depleted. We could infer that the four groups all decayed and the water in them was lost after 10 days of storage; in addition, there was no significant difference between different pretreatments in terms of the water-holding capacity of shrimps after a long period of chilled storage.

The close combination of water with other components in food can reduce the decomposition and deterioration caused by microbial growth and chemical reactions. Water activity (A_W_) is also an important quality index of sensory evaluation. The initial A_W_ values of the C, COS, CAP, and COS–CAP groups were all 0.992, while that of the COS–CAP group was significantly higher (0.93) on the 2nd day (*p* < 0.05). With the increase in storage time, the water activity of all groups decreased continuously, and the untreated group decreased the fastest, probably due to the continuous decrease in water content.

#### 3.2.2. TVB-N and pH Value Analysis

**Figure 5:**
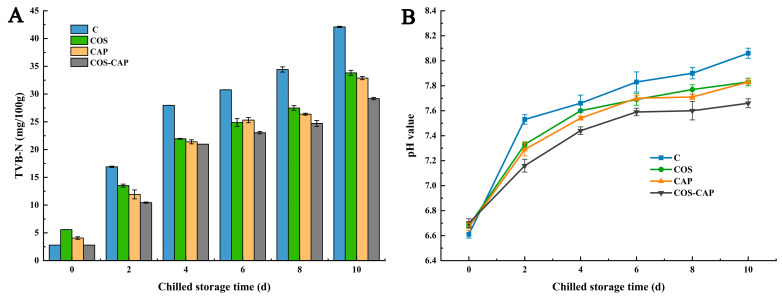
Changes in TVB-N content (**A**) and pH (**B**) of Pacific white shrimp during storage at 4 °C in control (C), 1% COS-treated group (COS), cold atmospheric plasma (50 kV, 4 min)-treated group (CAP), and 1% COS combined with CAP-treated group (COS–CAP).

TVB-N primarily consists of volatile ammonia and amines derived from the hydrolysis and breakdown of proteins. It is regarded one of the most significant indications of deterioration in aquatic products [35]. As illustrated in Figure 5A, the initial TVB-N values of the four groups were all inferior to 5.59 mg N/100 g. Relative to the control group, the growth in TVB-N values was dramatically decreased in COS, CAP, and COS–CAP groups from the 2nd day of chilled-storage (*p* < 0.05). Olatunde et al. [36] obtained a similar result for Asian sea bass slices. As shown in Figure 5A, the TVB-N values of all groups progressively increased, and the differences between treated groups were not obvious in the first 8 days. On the last day of the chilled-storage, the TVB-N values of the COS, CAP, and COS–CAP groups were 33.81, 32.88, and 29.18 mg N/100 g, respectively, which were significantly lower than control group (42.10 mg N/100 g). This significant difference in TVB-N value may possibly be due to the antibacterial effect of both COS and CAP. Based on the Chinese national standards for food safety of fresh and frozen aquatic products of animal origin (GB 2733-2015), we consider 30 mg/100 g as the limits of TVB content of shrimp. On day 10 of storage, the limit value was exceeded in all three groups except for the COS–CAP group. Relevant studies have confirmed that COS and CAP can both inhibit microorganism growth [37,38]. Therefore, treatment using COS in conjunction with CAP could effectively inhibit the accumulation of TVB-N in Pacific white shrimp during chilled storage.

A change in pH value can indirectly reflect muscle damage and protein changes. The pH value is considered to be directly related to the decomposition of shrimp and might be affected by the microorganisms and endogenous enzymes in shrimp muscle [39]. The pH value of shrimp samples exhibited an increasing trend over 10 days (Figure 5B). The increase in pH value may be due to protein breakdown and deamination under the action of microorganisms and enzymes. Generally, the pH value has a downward trend at the beginning; the decrease in pH is attributable to the growth and metabolism of LAB, which can create organic acids by metabolizing carbohydrates [40]. In this study, the pH value of shrimp did not decrease during chilled storage, probably because of the pH value having been minimized at or before storage, and then started to rise by 2 days of storage to above the initial value. The initial pH values of the control and treated groups were 6.61, 6.68, 6.67, and 6.70, respectively, similar to the pH value of fresh shrimp reported by Liu et al. [41]. Moreover, the pH value of samples treated with COS and CAP was relatively lower than those of untreated samples, probably because of the lower microbial abundance in treated samples. Thus, the decomposition of proteins, amino acids, and other nitrogenous substances into ammonia, trimethylamine, histamine, and other alkaline substances slowed down.

### 3.3. Sensory and Color Analysis

**Figure 6:**
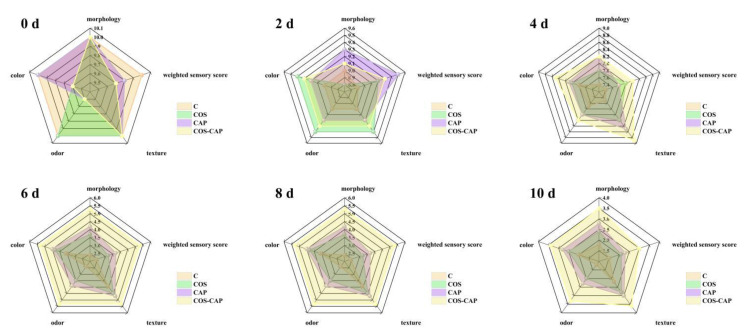
Changes in morphology, odor, color, texture, and weighted sensory score of Pacific white shrimp during storage at 4 °C in control (C), 1% COS-treated group (COS), cold atmospheric plasma (50 kV, 4 min)-treated group (CAP), and 1% COS combined with CAP-treated group (COS–CAP).

Sensory analysis aids physicochemical analysis in investigating the quality of aquatic products [21]. According to Qian et al. [35], the general appearance, odor, and texture were assessed to confirm the effect of combination treatment. In the same way, according to Zhao et al. [21], texture, odor, and color were evaluated to analyze the quality of large yellow croaker. Thus, sensory analysis can play an important role in quality evaluation. Changes in morphology, odor, color, texture, and the weighted sensory score of samples with different treatments stored in 4 °C are shown in Figure 6. The reduced initial odor value in the CAP and COS–CAP treatment groups may be due to the malodorous active substances such as ozone and nitrogen oxides produced by CAP. Significant reductions in properties including the color, odor, and texture of samples were observed as their storage duration increased. The decrease in the sensory score of Pacific white shrimp mainly manifested in two aspects. First, owing to the action of endogenous enzymes and exogenous microorganisms in the shrimp, the protein, amino acids, and other nitrogen-containing substances in the shrimp muscle were decomposed into ammonia, sulfur dioxide, trimethylamine, indole, and other metabolites [42], which caused the spoilage and bad odor of Pacific white shrimp. Second, the shrimp became black and red, and the meat softened. This is due to the role of enzymes in the shrimp. Polyphenol oxidase can oxidize the colorless compound monophenol into colorless bisphenol under aerobic storage conditions and then convert it into a highly active quinone substance. The substance is easily combined with protein or amino acid to produce melanin, and the shrimp body becomes black and red, making the senses unacceptable. In the end of the storage period, all four groups of samples reached sensory unacceptable values, but the COS–CAP group performed better than the control group in odor scores and other scores.

**Figure 7:**
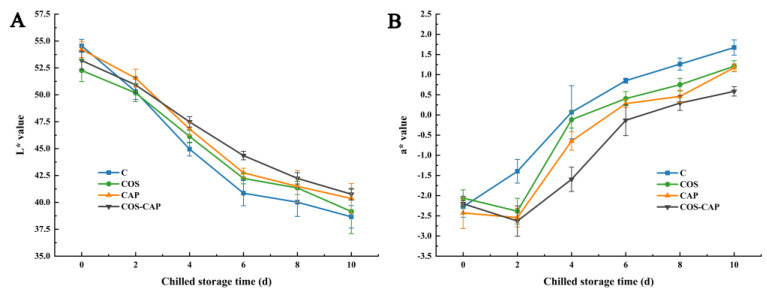
Changes in L* value (**A**) and a* value (**B**) of Pacific white shrimp during storage at 4 °C in control (C), 1% COS-treated group (COS), cold atmospheric plasma (50 kV, 4 min)-treated group (CAP), and 1% COS combined with CAP-treated group (COS–CAP).

The color, an external reflection of the aquatic products’ alterations in physiology and biochemistry, is frequently used by consumers to estimate quality [43]. In Figure 7A, it is shown that the L* value of fresh sample of shrimp was 54.56 and decreased during storage in four groups (*p* < 0.05), in agreement with the study of Shiekh [44]. The L* value of the shrimp samples treated using CAP and COS–CAP was lower compared to the fresh shrimp sample. Likewise, it is revealed in Figure 7B that the a* value showed an increasing trend during chilled-storage; the color of shrimp in all groups became more blackish or reddish, as characterized by their increased a* values and decreased L* values. Therefore, the COS–CAP-treated sample showed slow color changes, which may be due to the active species generated using CAP. According to some research, these reactive species are responsible for considerable chemical changes in the biomolecules of foods [45]. Previous research has revealed that the active species could result in pigment–protein complexes, which diminishes the whiteness of the surface as a result of the absorption of the pigment–protein [46]. In addition, the inactivation of enzymes was one of the most obvious modifications to dietary items brought on by plasma treatment of proteins [5], and the inactivation of polyphenol oxidase can efficiently maintain the black resistance of Pacific white shrimp.

### 3.4. Histological Changes

**Figure 8:**
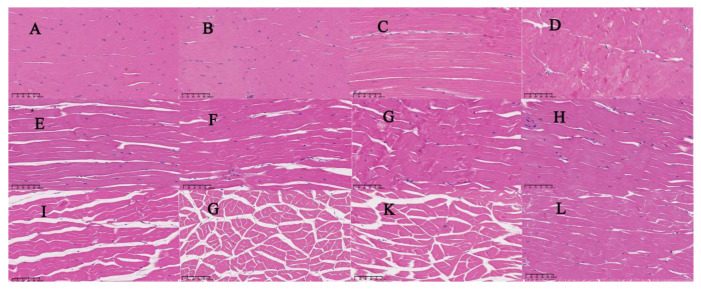
Changes in H&E staining micrographs of shrimp muscle tissue (transverse section) treated with COS, CAP, COS–CAP. (**A**–**D**) 0 d, (**E**–**H**) 4 d, (**I**–**L**) 8 d. (**A**,**E**,**I**)—untreated control (C); (**B**,**F**,**G**)—treated with COS (COS); (**C**,**G**,**K**)—treated with CAP (CAP); and (**D**,**H**,**L**)—treated with COS combined with CAP (COS–CAP).

According to histological analysis, the control group (Figure 8A) and treated group (Figure 8B–D) shrimp tissues exhibited normal morphology with a well-organized structure of good integrity and tightly connected myofibrils, having little space in between. There was no significant distinction between the four groups. After 4 days of chilled-storage, the myofibrils in the control group (Figure 8E) and the treatment group (Figure 8F–H) were significantly different, and the tissues of prawn were incoherent and somewhat disorganized. Moreover, comparing to the fresh samples, the extracellular spaces revealed a considerable expansion. Numerous larger extracellular spaces were formed between myofibrils, especially in the group of control muscle tissues on the 10th day (Figure 8I). Some myofibrils were severely damaged, forming many small fragments in the COS and CAP groups (Figure 8J,K). In contrast, the combined treatment group’s tissues showed few gaps and jarring myofibrils, which indicates good maintenance effects on the tissues’ physical characteristics. Notably, during 10 days of chilled storage, the histological changes in samples of the COS–CAP group were considerably less than for the control samples, in which myofibrils were seriously disrupted, which was also evident in the COS group samples and CAP group samples. These observations were consistent with the above sensory, physicochemical properties, and microbiological analysis.

### 3.5. Correlation Analysis between Physicochemical Changes and Microbiota

**Figure 9:**
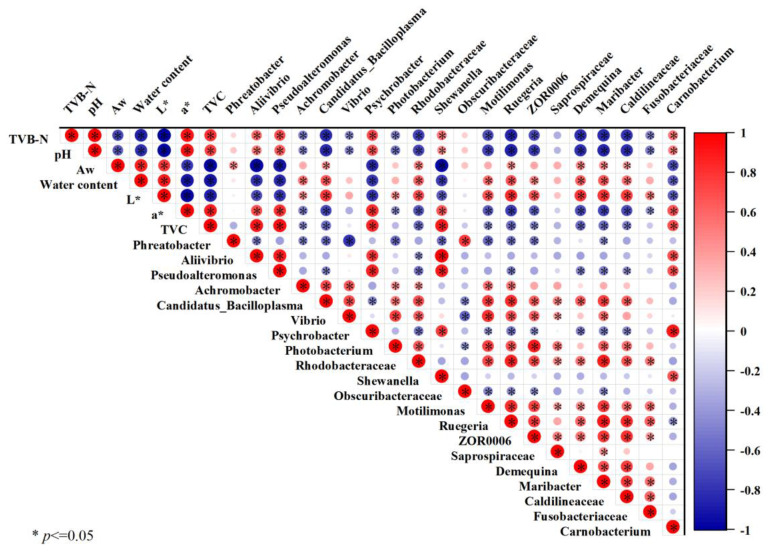
Pearson correlation analysis between microbiota composition of and physiochemical changes in Pacific white shrimp during storage at 4 °C.

The correlation heatmap (Figure 9), in which the red and the blue represent positive and negative correlation, respectively, showed the correlation analysis of the association between certain bacterial strains and environmental conditions. All indicators had strong relationships with each other and moderately high Pearson’s correlations. The value of TVB-N was positively correlated with the pH value, and both of them are positively correlated with a* value and TVC, while having significant negative correlations to A_W_, water content and L* value. In addition, *Aliivibrio* was positively correlated with the TVB-N, pH, a*, and TVC, while having significant negative correlations with water content and L* values. It also showed the same correlation in *Pseudoalteromonas*, *Psychrobacter*, and *Carnobacterium*. Moreover, there was a significant negative correlation between microbial indicators and physiochemical factors, including TVB-N, pH, a*, and TVC. It was revealed that microbial growth was one of the reasons for the deterioration of the abovementioned physiochemical factors. After treatment using COS–CAP, the growth of microorganisms decreased significantly. Thus, the significant weakening in the interaction among TVB-N, pH, and microorganisms may be the primary cause of the quality deterioration.

## 4. Conclusions

The results showed that CAP and COS treatment significantly inhibited the quality change of chilled shrimp and could prolong their shelf life. In particular, COS (1%) combined with CAP treatment largely affected the composition and quantity of microbiota in Pacific white shrimp during chilled storage, slowing down the increase in TVC, TVB-N, pH, and a*, as well as the decrease in water content, water activity, L*, sensory scores, and histological deterioration. In addition, further analysis of high-throughput sequencing revealed that there was a gradual decline in the microbial complexity over the period of chilled storage. At the conclusion of the storage period, *Aliivibrio* and *Pseudoalteromonas* dominated the bacteria in the sample of shrimp, and the treatment of combination of COS and CAP significantly slowed down the growth of dominant spoilage bacteria. Notably, neither COS nor CAP treatment exhibited obvious direct effects on the sensory properties of the shrimp. Notably, neither COS nor CAP treatments caused significant changes in the sensory properties of shrimp muscles. Therefore, both COS and CAP can be regard as alternative, nonthermal processing technologies for maintaining the physicochemical and sensory properties, as well as extending the shelf-life of chilled-storage shrimp. The combination of COS and CAP is superior in enhancing storage stability.

## Figures and Tables

**Table 1:** The 16S rRNA and ITS diversity index of the Pacific white shrimp during chilled storage (4 °C). Means ± SD were used to describe the results (n = 3). C = control group, COS = COS treatment, CAP = CAP treatment, COS–CAP = the combination of COS and CAP treatment.

Storage Time (d)	Sample	OTUs	Shannon	Simpson	Ace	Chao	Coverage
0	C	150.67 ± 10.97 Aa	2.96 ± 0.63 Aa	0.19 ± 0.13 Ad	154.78 ± 11.49 Aab	156.03 ± 9.61 Aabc	0.99
	COS	156.67 ± 25.48 Aa	3.05 ± 0.17 Aa	0.14 ± 0.07 Ad	159.75 ± 26.21 Aa	160.07 ± 27.68 Aab	0.99
	CAP	169.33 ± 6.66 Aa	2.76 ± 0.17 Aab	0.22 ± 0.05 Acd	174.61 ± 7.09 Aa	172.41 ± 7.23 Aa	0.99
	COS–CAP	115.00 ± 7.81 Bb	2.37 ± 0.54 Aabc	0.26 ± 0.14 Acd	120.25 ± 7.24 Bbc	122.46 ± 10.20 Bbcd	0.99
4	C	104.67 ± 34.56 Abc	2.14 ± 0.58 Abc	0.32 ± 0.10 Bcd	109.37 ± 34.74 Ac	117.78 ± 43.49 Acd	0.99
	COS	95.33 ± 3.79 Abc	1.74 ± 0.14 Acd	0.42 ± 0.04 Bbc	99.61 ± 7.45 Acd	98.03 ± 6.16 ABde	0.99
	CAP	71.67 ± 17.79 ABcd	1.37 ± 0.41 ABde	0.55 ± 0.11 ABab	72.70 ± 18.60 ABde	72.37 ± 18.54 ABef	0.99
	COS–CAP	38.00 ± 7.81 Be	0.75 ± 0.36 Be	0.70 ± 0.15 Aa	39.73 ± 23.07 Bef	41.83 ± 27.86 Bf	0.99
8	C	52.67 ± 4.16 Ade	1.85 ± 0.11 Acd	0.22 ± 0.02 Acd	57.07 ± 7.89 Aef	56.73 ± 8.40 Af	0.99
	COS	54.33 ± 15.04 Ade	1.76 ± 0.15 Acd	0.26 ± 0.04 Acd	56.94 ± 17.03 Aef	56.93 ± 17.42 Af	0.99
	CAP	29.33 ± 4.04 Be	0.72 ± 0.35 Be	0.70 ± 0.17 Ba	47.16 ± 11.93 Aef	39.33 ± 7.51 ABf	0.99
	COS–CAP	35.00 ± 2.65 Be	1.93 ± 0.04 Acd	0.21 ± 0.02 Ad	35.63 ± 2.65 Af	35.83 ± 2.75 Bf	0.99

Note: Values are provided in terms of mean ± SD, *n* = 3. The identical lowercase letters in a row and capital letters in a column both imply that there are no significant differences in either row or column (*p* > 0.05).

## Data Availability

The data presented in this study are available on request from the corresponding author.

## References

[1] Udayasoorian L., Peter M., Sabina K., Indumathi C., Muthusamy S. (2017). Comparative evaluation on shelf life extension of MAP packed *Litopenaeus vannamei* shrimp treated with natural extracts. LWT Food Sci. Technol..

[2] Sriket P., Benjakul S., Visessanguan W., Kijroongrojana K. (2007). Comparative studies on chemical composition and thermal properties of black tiger shrimp (*Penaeus monodon*) and white shrimp (*Penaeus vannamei*) meats. Food Chem..

[3] Nirmal N.P., Benjakul S. (2009). Effect of ferulic acid on inhibition of polyphenoloxidase and quality changes of Pacific white shrimp (*Litopenaeus vannamei*) during iced storage. Food Chem..

[4] Peng S.Y., Wei H.M., Zhan S.N., Yang W.G., Lou Q.M., Deng S.G., Yu X.X., Huang T. (2022). Spoilage mechanism and preservation technologies on the quality of shrimp: An overview. Trends Food Sci. Technol..

[5] Dharini M., Jaspin S., Mahendran R. (2023). Cold plasma reactive species: Generation, properties, and interaction with food biomolecules. Food Chem..

[6] Sruthi N.U., Josna K., Pandiselvam R., Kothakota A., Gavahian M., Mousavi Khaneghah A. (2022). Impacts of cold plasma treatment on physicochemical, functional, bioactive, textural, and sensory attributes of food: A comprehensive review. Food Chem..

[7] Koddy J.K., Miao W.H., Hatab S., Tang L.L., Xu H.I., Nyaisaba B.M., Chen M., Deng S. (2021). Understanding the role of atmospheric cold plasma (ACP) in maintaining the quality of hairtail (*Trichiurus lepturus*). Food Chem..

[8] Segat A., Misra N.N., Cullen P.J., Innocente N. (2015). Atmospheric pressure cold plasma (ACP) treatment of whey protein isolate model solution. Innov. Food Sci. Emerg. Technol..

[9] Zhou R.W., Rezaeimotlagh A., Zhou R.S., Zhang T.Q., Wang P.Y., Hong J.M., Soltani B., Mai-Prochnow A., Liao X.Y., Ding T. (2022). In-package plasma: From reactive chemistry to innovative food preservation technologies. Trends Food Sci. Technol..

[10] Gupta T.T., Ayan H. (2019). Application of Non-Thermal Plasma on Biofilm: A Review. Appl. Sci..

[11] Mourya V.K., Choudhari N. (2011). Chitooligosaccharides: Synthesis, characterization and applications. Polym. Sci. Ser. A.

[12] Muanprasat C., Chatsudthipong V. (2017). Chitosan oligosaccharide: Biological activities and potential therapeutic applications. Pharmacol. Ther..

[13] Xia W., Wei X.Y., Xie Y.Y., Zhou T. (2022). A novel chitosan oligosaccharide derivative: Synthesis, antioxidant and antibacterial properties. Carbohydr. Polym..

[14] Zou P., Yang X., Wang J., Li Y.F., Yu H.L., Zhang Y.X., Liu G.Y. (2016). Advances in characterisation and biological activities of chitosan and chitosan oligosaccharides. Food Chem..

[15] Choi B.K., Kim K.Y., Yoo Y.J., Oh S.J., Choi J.H., Kim C.Y. (2001). In vitro antimicrobial activity of a chitooligosaccharide mixture against *Actinobacillus actinomycetemcomitans* and Streptococcus mutans. Int. J. Antimicrob. Agents.

[16] Kim J.Y., Lee J.K., Lee T.S., Park W.H. (2003). Synthesis of chitooligosaccharide derivative with quaternary ammonium group and its antimicrobial activity against Streptococcus mutans. Int. J. Biol. Macromol..

[17] Chen H., Guo X.N., Zhu K.X. (2023). The effect of chitosan oligosaccharides on the shelf-life and quality of fresh wet noodles. Carbohydr. Polym..

[18] Sun T., Yao Q., Zhou D.X., Mao F. (2008). Antioxidant activity of N-carboxymethyl chitosan oligosaccharides. Bioorganic. Med. Chem. Lett..

[19] Tang L.L., Hatab S., Yan J.H., Miao W.H., Nyaisaba B.M., Piao X.Y., Zheng B., Deng S.G. (2022). Changes in Biochemical Properties and Activity of Trypsin-like Protease (*Litopenaeus vannamei*) Treated by Atmospheric Cold Plasma (ACP). Foods.

[20] Xu H.Q., Miao W.H., Zheng B., Deng S.G., Hatab S.M. (2022). Assessment of the Effect of Cold Atmospheric Plasma (CAP) on the Hairtail (*Trichiurus lepturus*) Quality under Cold Storage Conditions. Foods.

[21] Zhao Y.N., Lan W.Q., Shen J.L., Xu Z.F., Xie J. (2022). Combining ozone and slurry ice treatment to prolong the shelf-life and quality of large yellow croaker (*Pseudosciaena crocea*). LWT.

[22] Jiao L., Tu C.H., Mao J.L., Benjakul S., Zhang B. (2022). Impact of theaflavin soaking pretreatment on oxidative stabilities and physicochemical properties of semi-dried large yellow croaker (*Pseudosciaena crocea*) fillets during storage. Food Packag. Shelf Life.

[23] Jia S.L., Liu Y.M., Zhuang S., Sun X.H., Li Y., Hong H., Lv Y.M., Luo Y.K. (2019). Effect of ε-polylysine and ice storage on microbiota composition and quality of Pacific white shrimp (*Litopenaeus vannamei*) stored at 0 °C. Food Microbiol..

[24] Zhang B., Cao H.J., Wei W.Y., Ying X.G. (2020). Influence of temperature fluctuations on growth and recrystallization of ice crystals in frozen peeled shrimp (*Litopenaeus vannamei*) pre-soaked with carrageenan oligosaccharide and xylooligosaccharide. Food Chem..

[25] Liu C.S., Zhao D.F., Ma W.J., Guo Y.D., Wang A.J., Wang Q.L., Lee D.J. (2016). Denitrifying sulfide removal process on high-salinity wastewaters in the presence of *Halomonas* sp.. Appl. Microbiol. Biotechnol..

[26] Chen S.F., Zhou Y.Q., Chen Y.R., Gu J. (2018). fastp: An ultra-fast all-in-one FASTQ preprocessor. Bioinformatics.

[27] Salzberg S.L. (2011). FLASH: Fast length adjustment of short reads to improve genome assemblies. Bioinformatics.

[28] Edgar R.C. (2013). UPARSE: Highly accurate OTU sequences from microbial amplicon reads. Nat. Methods.

[29] Stackebrandt E., Goebel B.M. (1994). Taxonomic Note: A Place for DNA-DNA Reassociation and 16S rRNA Sequence Analysis in the Present Species Definition in Bacteriology. Int. J. Syst. Bacteriol.

[30] Wang Q., Garrity G.M., Tiedje J.M., Cole J.R. (2007). Naive Bayesian classifier for rapid assignment of rRNA sequences into the new bacterial taxonomy. Appl. Environ. Microbiol..

[31] Lan W.Q., Sun Y.Q., Liu S.C., Guan Y., Zhu S.Y., Xie J. (2022). Effects of ultrasound-assisted chitosan grafted caffeic acid coating on the quality and microbial composition of pompano during ice storage. Ultrason. Sonochemistry.

[32] Chen H.B., Wang M.Y., Yang C.F., Wan X.Z., Ding H.H.H., Shi Y.Z., Zhao C. (2019). Bacterial spoilage profiles in the gills of Pacific oysters (*Crassostrea gigas*) and Eastern oysters (*C. virginica*) during refrigerated storage. Food Microbiol..

[33] Poirier S., Rue O., Peguilhan R., Coeuret G., Zagorec M., Champomier-Verges M.C., Loux V., Chaillou S. (2018). Deciphering intra-species bacterial diversity of meat and seafood spoilage microbiota using gyrB amplicon sequencing: A comparative analysis with 16S rDNA V3-V4 amplicon sequencing. PLoS ONE.

[34] Zhang Y.T., Yao Y.J., Gao L.F., Wang Z.P., Xu B.C. (2018). Characterization of a microbial community developing during refrigerated storage of vacuum packed Yao meat, a Chinese traditional food. LWT Food Sci. Technol..

[35] Qian Y.F., Cheng Y., Ye J.X., Zhao Y., Xie J., Yang S.P. (2021). Targeting shrimp spoiler Shewanella putrefaciens: Application of ε-polylysine and oregano essential oil in Pacific white shrimp preservation. Food Control.

[36] Olatunde O.O., Benjakul S., Vongkamjan K. (2019). Combined effects of high voltage cold atmospheric plasma and antioxidants on the qualities and shelf-life of Asian sea bass slices. Innov. Food Sci. Emerg. Technol..

[37] Ansari A., Parmar K., Shah M. (2022). A comprehensive study on decontamination of food-borne microorganisms by cold plasma. Food Chem. Mol. Sci..

[38] Laokuldilok T., Potivas T., Kanha N., Surawang S., Seesuriyachan P., Wangtueai S., Phimolsiripol Y., Regenstein J.M. (2017). Physicochemical, antioxidant, and antimicrobial properties of chitooligosaccharides produced using three different enzyme treatments. Food Biosci..

[39] Cen S.J., Fang Q., Tong L., Yang W.G., Zhang J.J., Lou Q.M., Huang T. (2021). Effects of chitosan-sodium alginate-nisin preservatives on the quality and spoilage microbiota of Penaeus vannamei shrimp during cold storage. Int. J. Food Microbiol..

[40] Françoise L. (2010). Occurrence and role of lactic acid bacteria in seafood products. Food Microbiol..

[41] Liu W.J., Xie J., Li L., Xue B., Li X.H., Gan J.H., Shao Z.H., Sun T. (2021). Properties of phenolic acid-chitosan composite films and preservative effect on *Penaeus vannamei*. J. Mol. Struct..

[42] LI Y., Fang Y.D., Luo Y.K. (2018). Effect of ice coating on the quality of frozen shrimp (*Litopenaeus vannamei*). Meat Res..

[43] Cropotova J., Tappi S., Genovese J., Rocculi P., Dalla Rosa M., Rustad T. (2021). The combined effect of pulsed electric field treatment and brine salting on changes in the oxidative stability of lipids and proteins and color characteristics of sea bass (*Dicentrarchus labrax*). Heliyon.

[44] Shiekh K.A., Zhou P., Benjakul S. (2021). Combined effects of pulsed electric field, Chamuang leaf extract and cold plasma on quality and shelf-life of *Litopenaeus vannamei*. Food Biosci..

[45] Ucar Y., Ceylan Z., Durmus M., Tomar O., Cetinkaya T. (2021). Application of cold plasma technology in the food industry and its combination with other emerging technologies. Trends Food Sci. Technol..

[46] Gao S., Liu Y.Y., Fu Z.X., Zhang H.J., Zhang L.T., Li B., Tan Y.Q., Hong H., Luo Y.K. (2023). Uncovering quality changes of salted bighead carp fillets during frozen storage: The potential role of time-dependent protein denaturation and oxidation. Food Chem..

